# Improving inpatient postnatal services: midwives views and perspectives of engagement in a quality improvement initiative

**DOI:** 10.1186/1472-6963-11-293

**Published:** 2011-11-01

**Authors:** Debra E Bick, Val Rose, Annette Weavers, Julie Wray, Sarah Beake

**Affiliations:** 1 Kings College, London, Florence Nightingale School of Nursing and Midwifery, London UK; 2 Royal Berkshire NHS Foundation Trust, Reading, UK; 3 The University of Salford, School of Nursing and Midwifery, Manchester, UK

## Abstract

**Background:**

Despite major policy initiatives in the United Kingdom to enhance women's experiences of maternity care, improving in-patient postnatal care remains a low priority, although it is an aspect of care consistently rated as poor by women. As part of a systems and process approach to improving care at one maternity unit in the South of England, the views and perspectives of midwives responsible for implementing change were sought.

**Methods:**

A Continuous Quality Improvement (CQI) approach was adopted to support a systems and process change to in-patient care and care on transfer home in a large district general hospital with around 6000 births a year. The CQI approach included an initial assessment to identify where revisions to routine systems and processes were required, developing, implementing and evaluating revisions to the content and documentation of care in hospital and on transfer home, and training workshops for midwives and other maternity staff responsible for implementing changes. To assess midwifery views of the quality improvement process and their engagement with this, questionnaires were sent to those who had participated at the outset.

**Results:**

Questionnaires were received from 68 (46%) of the estimated 149 midwives eligible to complete the questionnaire. All midwives were aware of the revisions introduced, and two-thirds felt these were more appropriate to meet the women's physical and emotional health, information and support needs. Some midwives considered that the introduction of new maternal postnatal records increased their workload, mainly as a consequence of colleagues not completing documentation as required.

**Conclusions:**

This was the first UK study to undertake a review of in-patient postnatal services. Involvement of midwives at the outset was essential to the success of the initiative. Midwives play a lead role in the planning and organisation of in-patient postnatal care and it was important to obtain their feedback on whether revisions were pragmatic and achieved anticipated improvements in care quality. Their initial involvement ensured priority areas for change were identified and implemented. Their subsequent feedback highlighted further important areas to address as part of CQI to ensure best quality care continues to be implemented. Our findings could support other maternity service organisations to optimise in-patient postnatal services.

## Background

Improving healthcare performance is an increasing challenge globally. High quality service provision and enhanced patient experience are a common element of healthcare policy in many industrialised countries, including within the United Kingdom maternity services [1]. Despite experiences of care in UK National Health Service (NHS) hospitals immediately following birth frequently being reported by women as negative [2-5] there has been limited work to address how the acute sector services could improve this aspect of care.

In the UK, midwives provide the majority of care for women during and after their pregnancies. Although the majority of women in the UK give birth in hospital, midwives' views of the value and role of the postnatal care they provide has received little attention, despite being a core element of the midwifery role since the early twentieth century [6]. One small early study reported that midwives saw little value in the routine observations and examinations of maternal physical recovery they undertook as part of routine postnatal contacts [7]. A large UK cluster trial of protocol based midwifery led postnatal care found that midwives continued to undertake routine physical examinations and observations at each contact despite guidance on planning care based on individual need, rather than routine [8]. A state-wide review of postnatal care in Victoria, Australia found that the views and experiences of midwives were similar to those of the postnatal women, in that they were less satisfied with the organisation and provision of hospital postnatal care than care in the community and perceived a need for individualised, unrushed, flexible care [9].

Over the last two decades there has been a constant drive to introduce change into the UK maternity services against a backdrop of finite resources, an increasing birth rate and concerns about the poorer health of women who become pregnant [1,10-12]. Drivers include health service improvement targets to increase 'best' practice outcomes, including the duration of breastfeeding [13]. There has also been a focus on reducing variations in health outcomes through use of guidelines and standards to increase evidence based practice [11], and recommendations to enhance the safety and quality of care [14,15]. Drivers to improve maternity care have been prompted by an increase in patient complaints and number of adverse events, including an increase in the number of UK women who have a postpartum haemorrhage [16]. Greater financial pressures are also impacting on service provision [17] and need for more efficient services. Despite the policy agenda to introduce service revisions to improve health care outcomes [18], little has been published on the use of quality improvement as an approach to enhance the content and provision of maternity care or to disseminate how others can learn from the experiences of maternity teams who have successfully implemented change.

Since the 1990s, many sectors of the UK NHS have adopted change management models and tools to improve service outcomes, more often informed by theories developed in industry [19], including Total Quality Management (TQM), Lean Thinking, Continuous Quality Improvement (CQI) and Six Sigma [20]. To date, there has been little consistency in the content of quality improvement programmes, with organisations using a range of approaches and tools [20]. Many models are prescriptive, identifying different phases or steps to introduce change, using either 'linear' or 'cyclical' approaches. However, prescriptive models may disregard the complexity of change within the health care environment and not take into consideration issues which may arise from the impact of change on clinical staff. There are also more analytical approaches to clinical quality improvement which do not include a standard model or take the complexity of change into account [21]. Within the UK maternity services, quality improvement initiatives have frequently been initiated by professional organisations, for example, the Royal College of Midwives' campaign to increase normal birth [22]. Whilst these are useful initiatives and raise the profile of priorities where change to improve service delivery and outcomes is needed, the improvement agenda is still reliant on local adoption, implementation and evaluation.

Powell et al [20] produced a systematic narrative review, commissioned by NHS Quality Improvement Scotland, which focused on developing an understanding of the approaches available to drive quality improvement, their strengths and weaknesses when applied to healthcare, and potential for implementation in healthcare settings in Scotland. The review highlighted particular challenges for health care organisations when introducing quality improvement initiatives. These included the need to reflect the complex care processes involved; the role and contribution of multiple stakeholders; long-standing inter-and intra- professional 'turf wars'; an emphasis on individual proficiency rather than team-working and a history of challenging relationships between managers and health professionals' which may interrupt successful implementation of change. The authors identified that a broad set of *'necessary, but not sufficient' *[p5] conditions were required for successful implementation. These included: availability of practical and human resources to enable quality improvement; the active engagement of health professionals; sustained managerial focus and attention; multi-faceted interventions; coordinated action at all levels of the organisation; substantial investment in training and development; and the availability of robust and timely data through supported IT systems [20].

This paper presents data on the views of midwives from one large maternity unit in the South of England following the introduction of an organisation wide quality improvement initiative to improve in-patient postnatal care and processes to transfer women home. The initial work was informed by several drivers for change. The senior hospital management team wished to develop care in line with National Institute for Health and Clinical Excellence (NICE) guidance for postnatal care [23] and to improve their hospital based postnatal services in response to concerns from service users about the quality of inpatient care. Other drivers included a lower than national average breastfeeding uptake rate among women giving birth at the unit. From the outset, the quality improvement work engaged not only midwives working across the different clinical areas, including the community, but also other stakeholders including obstetricians, senior clinical leads, representatives of local service users, finance and facilities leads. As midwives were the largest clinical group consulted as part of this process, whose activities were most likely to be directly affected, this paper reports on their views of the change process and their roles within this. As Ovretveit [24] highlighted, as quality improvement initiatives are highly influenced by the context into which they are introduced and by the processes of implementation, our findings may support others to address how clinician engagement could be enhanced.

## Methods

The study took place at one district general hospital in the South of England with around 6,000 births a year. As the intention was to introduce change across the organisation to enhance in-patient care and discharge home, with a focus on systems and process change, a model which would best support this approach was needed. A continuous quality improvement (CQI) approach was selected as the most appropriate to inform the QI work at the study site, which was introduced over a 10 month period. The CQI model views quality improvement as an ongoing activity, integrated within the organisation, emphasises the role of senior management engagement with project teams, and places importance on measurement [20,25].

### Planning the improvement initiative

The initiative followed a number of steps informed by a CQI approach to identify where change could be achieved across the organisation to promote better preparation of women for their postnatal recovery and transfer. The study team obtained perspectives of a wide range of stakeholders on their perceptions of the barriers and facilitators to effective postnatal care in hospital including those of the women [26], clinicians and senior clinical managers in midwifery and obstetrics. Two focus groups were held with midwives from across the acute unit and community teams, and six interviews took place with individuals in senior clinical management roles. In addition to seeking the views of stakeholders, process mapping of the 'journey' for postnatal women through the organisation to identify 'bottle-necks' in the system following different modes of birth was carried out by a multi-disciplinary team comprising senior midwives, obstetricians, practice educators and the unit modernisation team. It was also important to address how the postnatal care of women was documented to reflect compliance with Standard 5 of the Clinical Negligence Scheme for Trusts risk management protocol for maternity services [27]. Work with the management and clinical governance teams took place to review and revise the maternal and neonatal postnatal records.

### Content of the improvement initiatives

Following the preparatory development work, changes implemented across the organisation included the piloting and introduction of new handheld records to prompt evidence based individualised care in line with NICE guidance [23]. This included an emphasis on the need to only undertake routine physical observations and examinations after the first postnatal contact based on the women's individual needs. At the request of the clinical governance team, the new records included a Maternity Early Obstetric Warning Score (MEOWS) chart in line with the recommendation of the previous Confidential Enquiry [12] and the Waterlow Scale to assess risk of pressure ulcer development [28].

Hospital postnatal discharge routines were revised to promote practice in line with NICE guideline recommendations [23]. These included stays on delivery suite of up to three hours post vaginal birth to encourage skin-to-skin contact and initiation of breastfeeding. Postnatal discharge preparation commenced on delivery suite, with midwives asked to complete computer records for women requesting early hospital discharge. Following our initial work with women interviewed on the postnatal wards [26], a range of sources of information for parents on aspects of infant care were introduced onto the wards, including daily infant bathing demonstrations organised by maternity support workers, a range of breastfeeding information including posters on the wards, a leaflet to introduce women to the postnatal ward, and changes to the processing and issuing of routine prescriptions for pain relief and other medication women would need to take home with them. As part of the change process 18 half day workshops were held, attended by over 100 clinical staff, mainly midwives and maternity support workers, to discuss the planned changes to care systems and processes, to explain the new postnatal notes, explore the importance of effective communication and provide an opportunity for discussion.

### Content of the questionnaire

To address the midwives' views of the impact of revisions to postnatal care on their roles and explore their level of engagement with the quality improvement processes, including the preparatory phases, midwives were surveyed following the 10 month implementation period. A questionnaire specifically developed for the study, included mostly closed questions which required a 'yes' or 'no' response, or a response to five item Likert scales. The midwives also had the opportunity to comment using open text on their responses to many of the questions. Midwives were asked to complete all sections of the questionnaire.

The questionnaire was piloted on six midwives, following which minor amendments to the format of some questions were made. As midwives are responsible for the planning and supervision of postnatal care, they were the only professional group targeted for this stage of the study. The questionnaire was divided into three parts to reflect changes to each stage of a woman's process through the postnatal system. Questionnaires were distributed to midwives via the internal hospital post. It was not possible at the outset to ascertain how many midwives would be eligible to complete the questionnaire, as a number of new appointments and revisions to midwifery staff rotas had been introduced. To be eligible to complete a questionnaire, the midwife had to have been in post at the unit at the commencement of the project and had to be involved in the provision of postnatal care. Senior midwifery managers and midwives who only provided antenatal care were excluded. A box was placed on each ward for the questionnaires to be returned. A reminder to complete the survey was circulated after two weeks.

### Data entry and analysis

Quantitative data were entered and analysed to present descriptive statistics using an Excel data package. Open comments in response to questions were transcribed separately and quotes selected to provide context to support midwives views of the quality improvement changes.

### Ethical approval

Ethical approval was obtained from Berkshire Research Ethics Committee (REC reference number 07/H0505/124). No identifiable data were collected from any of the respondees.

## Results

### Response rate and baseline data

At the time of the survey 178 midwives were in post at the unit. It was estimated that of these, 149 midwives were eligible to complete the questionnaire as they were involved in some aspect of postnatal care, 68 (46%) of whom responded. Eighteen midwives worked mostly on the postnatal ward at the time of completing the questionnaire, 25 were working on the labour ward and 25 were working in the community. All of the midwives were female. Two thirds were aged 40 years or older and two-thirds had been qualified as a midwife for 10 or more years. Forty (59%) midwives were employed on a band 6, and 27 (40%) as a band 7 with one midwife at band 8a (all UK NHS non-medical posts have set salary scales (or 'bands') which range from 1 to 9, with salaries based on a nationally agreed job evaluation scheme). Most midwives are on pay bands 6 to 8. Six (9%) midwives had a masters' degree. The following sections present the midwives' responses.

### Revisions to the organisation of care on the postnatal ward

All of the midwives were aware of the quality improvement work. Under half were aware of the work through attendance at the training workshops (29/43%), ad hoc meetings with the research midwife (25/38%) or through other unit meetings (11/16%). Other sources of information about the project, included seeing posters distributed through the unit, seeing copies of the pre-intervention questionnaires handed to women on hospital discharge, e-mails and discussions with project team members. When asked if they had received adequate information to support revisions to care on the postnatal ward, 30 (44%) of midwives felt they had, while 8 (14%) did not and 18 (32%) did not know.

Over two thirds of the midwives' felt that the revisions to the content of care on the postnatal wards were more appropriate to meet the women's physical and psychological health needs, and their information and support needs (Table 1). Over a third (30/44%) were 'very' or 'fairly' satisfied with the revisions to care processes on the postnatal ward.

**Table 1:** Midwives views of revisions to the planning and content care on the postnatal ward

Did revisions improve care?	Yes N (%)	No N (%)	Don't know N (%)	Total responses N (100%)
Women's individual physical health	33 (65)	1 (2)	17 (33)	51

Women's individual psychological health	33 (60)	3 (5)	19 (35)	55

Parent's information needs	32 (67)	2 (4)	14 (29)	48

Parent's support needs	29 (60)	6 (12)	13 (27)	48

Use of midwives' skills	27 (49)	8 (14)	20 (36)	55

Use of midwives' time	21 (39)	14 (26)	19 (35)	54

When asked if the revisions to the current systems and processes made more appropriate use of their time, 21 (39%) midwives, including six who worked in the community, stated that they had, however 14 (26%) midwives did not feel this was the case, and 19 (35%) did not know.

When asked which specific aspects of revisions to care on the postnatal ward they considered had made the biggest difference to women's experiences, practical support for parenting, for example, introducing infant bath demonstrations on the ward, were cited most often (12/18%), followed by introduction of the new postnatal notes (7/10%). The following quotes illustrate the midwives views:

'They (parents) really enjoy and are reassured by watching a baby bath' (midwife on postnatal ward)

and

'baby care definitely helps with mothers psychological health' (midwife on postnatal ward)

### Revisions to postnatal care on delivery suite

Over two thirds of the midwives (42/61%) felt that the revisions to immediate postnatal care on the delivery suite were more appropriate to assess the individual health and other needs of the woman. When asked what revisions had made most difference, 26 (38%) midwives responded that allowing more time on delivery suite prior to ward transfer supported women to have uninterrupted skin-to skin contact with their infant and initiate breastfeeding. Quotes to support this included:

'Breastfeeding and skin-to-skin care made more of a priority. Problems picked up earlier' (midwife on delivery suite)

and

'Allowing time with baby - not having to hurry mum' (midwife on delivery suite)

Over half (36/53%) of the midwives who completed the Likert scale on satisfaction with revisions to care were either 'very' satisfied or 'fairly' satisfied with the revisions to routine systems and processes introduced into the delivery suite, with around a quarter (14/26%) neither satisfied nor dissatisfied and three midwives fairly dissatisfied.

When asked whether the revisions to the transfer of women from the delivery suite to the postnatal ward made more appropriate use of their skills, just under half (31/45%) thought they did, and over a third (25/37%) thought they made more appropriate use of their time. Thirty midwives (44%) felt that they had received adequate information to support the revisions to care on delivery suite. Only two midwives reported that they did not.

### Introduction of the new postnatal records

When the midwives were asked if they were aware that new postnatal records had been introduced, not unsurprisingly given their introduction across the unit, the majority (91%) were aware. Over a third (26/38%) were first made aware of the records through the training workshops, a quarter (18/26%) from meetings with the research midwife, a quarter (17/25%) by seeing the new notes in use and 15 (22%) through attendance at unit meetings. Other ways mentioned by the individual midwives in response to an open question included at routine ward hand over meetings, posters advertising the improvement project, piloting of the new notes prior to general introduction, and on staff notice boards.

When asked if the new postnatal records helped them to better plan and implement care, three-quarters (49/73%) of the midwives said that the notes supported better planning of care to meet women's *physica*l health needs (Table 2), while around two thirds felt they supported planning to meet a woman's *emotional *(42/63%) and *social *needs (39/58%). Lower responses were achieved in terms of the new notes informing improved infant feeding support, with 28 (41%) midwives replying positively, and 19 (28%) negatively.

**Table 2:** Midwives views of introduction of new postnatal records

Do the new postnatal records support better planning of care?	Yes N (%)	No N (%)	Don't know N (%)	Total responses N (100%)
Better planning & carrying out of care to meet women's physical health needs	49 (73%)	8 (12%)	10 (15%)	67

Better planning & carrying out of care to meet women's social needs	39 (58%)	14 (21%)	14 (21%)	67

Better planning & carrying out of care to met women's emotional health needs	42 (63%)	10 (15%)	15 (22%)	67

Improved infant feeding support	28 (41%)	19 (28%)	21 (31%)	68

Fifty four (80%) midwives found having the MEOWS chart in the notes helpful. When asked why, it was reported that the tool made it easier and quicker to identify any deviations from a woman's expected postnatal recovery. In contrast, only 28 (41%) midwives found inclusion of the Waterlow assessment tool helpful.

The most common theme identified from the open questions with respect to the new notes was that they resulted in more 'paper work'. Although many midwives commented positively, stating they liked the style with the potential for care to improve as a consequence, in practice they found them time consuming to complete. In response to the open question, it was apparent that it was the time needed to capture the additional information required to plan care which was the issue, for example

'More paperwork, more questions to go through with women' (midwife, postnatal ward)

and

'If you give all the information asked for in the booklet it does take time' (midwife, postnatal ward)

One aspect that contributed to the extra time was if the previous midwife who had cared for a woman had not completed all relevant sections of the notes. Several community midwives commented that some hospital midwives did not complete the sections on the women's obstetric history adequately. As a consequence their first home contact took longer as they had to ask the women about this. Nevertheless, there were perceived benefits, as the following quote illustrates:

*I think the notes are excellent; they cover every aspect of postnatal care and act as a prompt for less experienced staff. However it takes between 40 minutes and one hour to complete at the first community visit (community midwife)*.

### Revisions to the content of midwifery postnatal care for individual women

Midwives were asked what routine postnatal observations and examinations they performed and when they would perform them in relation to the time since the birth (Table 3). As the new postnatal records reflected NICE guidance, the aim was to assess if midwives were able to individualise routine observations and examinations according to the needs of a woman. At most contacts (which could include ward or community contacts) over three-quarters of the midwives (52/78%) said they would measure uterine involution, 60 (89%) would assess vaginal loss (lochia), 55 (82%) would examine the woman's legs and 52 (78%) midwives reported they would observe a woman's perineum at each contact. A third of the midwives would also check the woman's temperature. Few midwives reported that they would only perform these observations and examinations at the first visit. When asked what other observations they would undertake at each contact, two midwives said they would ask about emotional well being and two midwives said they would ask about pain relief needs. Individual midwives mentioned that they would ask about a woman's bladder and bowel function at each contact.

**Table 3:** Timing of when midwives would perform observations and examinations

When would you perform the following observations and examinations?	At first contact only N (%)	At most contacts N (%)	Only when necessary N (%)	Total responses N (%)
Measurement of uterine involution	6 (9%)	52 (78%)	9 (13%)	67

Blood pressure recording	9 (13%)	25 (37%)	33 (49%)	67

Assessment of lochia	5 (7%)	60 (89%)	2 (3%)	67

Breast examination	8 (12%)	37 (56%)	21 (32%)	66

Temperature	7 (10%)	22 (33%)	38 (57%)	67

Legs	7 (10%)	55 (82%)	5 (7%)	67

Perineum	7 (10%)	52 (78%)	8 (12%)	67

### Overall views of revisions to postnatal care

When asked if women's health needs had benefited as a result of the quality improvement work, 53 (85%) of the 62 midwives who responded to this said 'yes a lot' or 'yes a little,' and in response to whether women's support needs had benefited, 54 (87%) said either 'yes a lot' or 'yes a little'.

When asked if the revisions to postnatal care had increased their workload, 63 (93%) of the 68 midwives who replied said 'yes a lot' or 'yes a little'. For most this was as a consequence of the extra paper work generated by the new notes. Most felt that they had received sufficient help with the revisions to routine systems and processes, through attending staff training sessions and regular contact with the research midwife in the clinical areas. The midwives were also asked to indicate from a given list where they felt further revisions to care were required to continue to improve postnatal care. For the majority, more midwives and clerical staff were the most important factors (Table 4)

**Table 4:** Midwife views of further changes required to improve postnatal care

Item	Total 68 N (%)
More midwives (postnatal ward)	58 (85%)
More midwives (delivery suite)	57 (84%)
More clerical staff	49 (72%)
Better communication between maternity staff	43 (63%)
More maternity support workers (postnatal ward)	42 (62%)
More maternity support workers (delivery suite)	38 (56%)
Better antenatal education for parents	28 (41%)
Better training to support breastfeeding	20 (29%)
Longer in-patient stay on postnatal ward	19 (28%)
Regular updating of clinical postnatal skills	15 (22%)

## Discussion

At the core of this quality improvement initiative was recognition of the need to improve women's experiences of transfer through the hospital after giving birth and then onto home, and support implementation of best practice evidence to enhance maternal and infant health outcomes. There is a dearth of information to support effective transfer of postnatal women from hospital to home, other than in relation to evidence of no impact of early compared with later hospital discharge [29]. However there is a growing evidence base of the impact on a range of outcomes following transfer of a woman from home to hospital during labour [30] and the impact on women's satisfaction with childbirth on being transferred from one model of maternity care to another [31]. Introducing change in health care settings is complex and maternity care which is funded and organised as part of an acute medical unit is no different to other settings in this respect, given the multiple stakeholders involved. Our findings could support others working in the maternity services to enhance service users' experiences and clinical outcomes through consideration of how to engage staff in quality improvement initiatives at a time when increasing pressure is being placed on finite health service resources. Process outcomes and the views of the clinical staff involved in change initiatives are rarely reported, leaving a gap in learning opportunities.

The active engagement of health professionals is one of the conditions which need to be in place to support implementation of organisational change, regardless of the method of quality improvement adopted [20]. In this project the obstetricians and midwives were consulted and involved from the outset. As the obstetricians' involvement in routine postnatal care is minimal, the main focus of engagement once the quality improvement work was underway was with the midwifery teams. Use of multi-faceted interventions and co-ordinated action at all levels of the health care system with regular feedback to the managers was also a priority for our work [20]. The adaptation of a CQI model worked well for our study in terms of clinician engagement with the systems and process changes they were contributing to as part of their daily working practices [32].

The main limitation of the current evaluation is the low response rate, with the potential that findings may not represent the views of the midwifery staff across the organisation as a whole. Ensuring all midwives who were eligible to participate received a copy of the questionnaire was difficult as many worked across the health sectors, and some only worked part-time. Despite this, we achieved good representation of midwifery staffing grades and representation of those working across the primary and secondary care sectors. MacArthur et al [8] in their large cluster trial of a new model of postnatal midwifery-led community care obtained an overall response rate of 70% from a survey of the participating midwives, however the sample comprised midwives linked to general practice 'clusters', who were in regular contact with the trial team to promote adherence to trial processes, unlike the current study where the midwives may not have had direct contact with a member of the study team. Evaluations of future quality improvement initiatives in maternity care may be better to consider a range of approaches to capture the views of key clinical staff.

Despite the relatively low response rate, it was apparent that the planning and preparation undertaken to engage midwives with the implementation of the revisions to routine care was successful. The majority were aware of changes introduced, although we acknowledge that the converse could have been the case for those who did not respond. The midwives generally considered that the revisions to the content of care on the postnatal wards were more appropriate to meet women's physical and emotional health, information and support needs.

Of note is that some midwives perceived particular benefits from the introduction of practical infant care demonstrations on the wards. Perceived benefits included the potential to save time during community contacts, and importantly, enhanced parent well-being and reassurance. Infant care demonstrations within routine postnatal in-patient care could support new parents to develop confidence to take care of their babies, and provide an opportunity for clinical staff to discuss the importance of parents interacting with their infant. A recent Cochrane review of postnatal parental education interventions for optimizing infant general health and parent-infant relationships found insufficient evidence to determine the effects of these [33]. However of the 15 trials included in the review, only one was from the UK and interventions up to two months post birth were included. Further research is needed to evaluate the potential benefits of providing practical infant care demonstrations as part of routine hospital based postnatal care.

Although the main focus of the project was to enhance women's experiences of inpatient postnatal care and transfer home, it was clear from the preparatory work that systems and processes across the continuum of pregnancy and birth would have to be addressed as all could potentially impact on postnatal care. This included revising routine processes on the delivery suite in line with evidence to support skin to skin care and uptake of breastfeeding [23], and completing documentation for women who required early transfer home. It was encouraging that midwives also reported that revisions to care were of benefit for women, and made better use of their skills and time.

One aspect of the initiative midwives found unhelpful was the additional workload the new postnatal record generated, although this was hard to quantify. There were also some perceived benefits of introducing the new notes, including better support for less experienced staff. The previous notes used at the unit did not require midwives to document a care plan for the woman and her infant or provide guidance on details of care to be covered at specific time periods [23]. It is possible that once the midwives were used to the new records, these issues would resolve. Another option may be to consider introducing a set of notes for the whole maternity episode, which could reduce duplication.

It is well recognised that patient safety in the general population can be compromised by poor hospital discharge summaries, poor inter-professional communication and lack of appropriate co-ordination [34,35]. The rapid discharge of women from hospital following birth, which more likely than not involved clinical intervention, could compromise postnatal recovery if insufficient attention is paid to how transfer is planned and managed. The most common cause of adverse events in patient care is a failure in care communication and coordination [36]. It was anticipated that the new postnatal records would promote more effective and efficient communication between clinical teams and between the individual clinician and the woman. Despite attendance at workshops, other sources of information about the new notes and feedback that the improvement changes enhanced midwifery skills and time, some midwives were concerned that colleagues were not completing information as required. This is not just an issue for the handover of a woman from acute to primary care during the postnatal period, as similar issues have been identified with respect to the documentation of the assessment and management of perineal trauma by midwives [37,38]. Given the increased focus on litigation in maternity care, more attention should be directed to ensuring all clinicians are aware of the importance of accurate record keeping [27].

One of the key recommendations of recent Confidential Enquires into Maternal Deaths [12,39] is that a Modified Early Obstetric Warning System (MEOWS) should be introduced into all clinical areas where a pregnant or postnatal woman may be admitted. MEOWS are adapted from Modified Early Warning Scores (MEWS) developed for general population use. The MEWS system uses five physiological parameters to calculate a score: respiratory and heart rates, temperature, level of consciousness and systolic blood pressure. Each parameter has a value, and the sum of these produces a total score. To date, outcomes of the use of MEOWS in maternity care settings have not been assessed, although there is evidence from a retrospective study of women who had intrauterine infection at one clinical site in the United States that MEWS developed for general population patients should not be used in an obstetric population [40].

Of interest is that most of the midwives were positive about the inclusion of a MEOWS tool, although the tool did not include signs or symptoms indicative of escalating illness in a postnatal woman (for example, offensive lochia [41]), and was not validated for use in this group of women. The tool required a score for each parameter measured, with anecdotal evidence that some midwives were not calculating an accurate overall score or only measuring certain parameters, for example a woman's blood pressure. The midwives reported that the MEOWS made it easier to identify any deviation in a woman's recovery from the birth. Whether the identification of maternal observations outside of normal parameters trigged an appropriate response was not an aim of the survey, however it should be investigated further.

The inclusion of the Waterlow Pressure Ulcer Risk Assessment scale was requested by the study site clinical governance directorate, following a small number of reported cases of women developing pressure sores in labour. For the midwives, the tool had little relevance for their clinical care. There is no scale validated for use in an obstetric population, and the Waterlow scale which includes data collection categories on gender and older age, is clearly not appropriate for women of childbearing age. Some groups of women are acknowledged to be at risk of developing a pressure sore, including those who are obese or receiving high dependency care, however it is not known if units generally document the incidence and prevalence of this level of morbidity. Single case reports of pressure ulcer development in the sacral and heel areas have been reported following use of epidural analgesia [42]. If this is an area of concern which should be a focus for midwives, work with tissue viability and other experts in pressure area care is clearly warranted to develop identification and appropriate management pathways [43].

Despite guidance that routine postnatal observations and examinations should be based on individual need [23], the majority of midwives reported that they would perform these at each contact, supporting the earlier finding of MacArthur et al [8]. There could be several reasons for this, including midwives' confidence in moving away from a traditional content of care as well as refocusing the planning and content of postnatal contacts on a more holistic approach to maternal physical and psychological health needs. The midwives clearly felt further revisions were required to improve postnatal care in hospital, most commonly that additional midwives and maternity support workers were required. It is possible that revising routine systems and processes to identify barriers to streamlining effective care could improve women's experiences without the need for more clinical staff. A review of systems and processes should be considered as a priority first step to identify how care delivery could be supported to reflect best evidence. Despite the huge drive to implement evidence based practice to close the evidence to practice 'gap' [44], if the systems supporting and informing current care processes are not addressed, it is unlikely evidence will become sufficiently 'embedded' to influence outcomes as intended. As Glasziou and colleagues [44] highlight evidence based medicine which focuses on 'doing the right things' and quality improvement which focuses on 'doing things right' should be viewed as complementary, with benefits for patients if these approaches were integrated. Increasing the midwifery complement may only be one part of the 'jigsaw' of improving care to reflect evidence of benefit within the maternity services.

A recent evaluation by the National Childbirth Trust, a large UK consumer group, of first-time mothers' experiences of postnatal care [5] found little improvement in perceptions of hospital care in the ten years since a previous survey by the same organisation [3], despite the introduction of NICE guidance [23]. Barriers to change in maternity care have been attributed to staff who are stressed and consequently resistant to change, as well as poor management and financial restraints [45]. Although successful approaches to improving care in an acute medical environments may not necessarily be applicable in a postnatal care setting as the context of care is so different, it is just as imperative that improving care is viewed as an intrinsic part of the day to day role of all relevant stakeholders [46].

## Conclusions

Whatever quality improvement approach is used when introducing change into maternity care, it is important to involve all relevant stakeholders from the outset. Midwives play a leading role in postnatal care and their involvement at all stages in this work, from the identification of barriers to system performance to feedback on the pilot version of the new postnatal records to attending workshops was crucial to the success of this project. It was also imperative that the rationale for introducing changes to routine systems and processes was viewed by the midwives as promoting a continuum of effective pregnancy and birth care. Following the introduction of the changes to care systems and processes, it is as important to assess the impact of these on the staff responsible for implementing the changes. The midwives were on the whole positive with the outcomes in terms of the impact on the women as well as their own practice.

Continuous quality improvement is an evolving process and following on from the revisions to systems and processes described here, further improvements are planned based on experiences to date. Our approach which considered the systems and processes of care, the documentation and measurement of care and clinician development through workshops and regular updates on progress may serve as a useful approach for others wishing to improve aspects of their maternity care provision.

## Competing interests

The authors declare that they have no competing interests.

## Authors' contributions

DB conceived the study, was lead investigator, participated in its design and data analysis and helped to draft the manuscript. SB participated in the data analysis and coordination of the study and drafted the manuscript. VR collected the data and contributed to the manuscript. AW and JW contributed to the design of the study and the manuscript. All authors read and approved the final manuscript.

## Pre-publication history

The pre-publication history for this paper can be accessed here:

http://www.biomedcentral.com/1472-6963/11/293/prepub
